# Pitfalls in the Diagnosis of Posterior Circulation Stroke in the Emergency Setting

**DOI:** 10.3389/fneur.2021.682827

**Published:** 2021-07-14

**Authors:** Carolin Hoyer, Kristina Szabo

**Affiliations:** Department of Neurology and Mannheim Center for Translational Neuroscience, University Medical Center Mannheim, Mannheim, Germany

**Keywords:** emergency department, diagnostic error, misdiagnosis, stroke, posterior circulation

## Abstract

Posterior circulation stroke (PCS), caused by infarction within the vertebrobasilar arterial system, is a potentially life-threatening condition and accounts for about 20–25% of all ischemic strokes. Diagnosing PCS can be challenging due to the vast area of brain tissue supplied by the posterior circulation and, as a consequence, the wide range of—frequently non-specific—symptoms. Commonly used prehospital stroke scales and triage systems do not adequately represent signs and symptoms of PCS, which may also escape detection by cerebral imaging. All these factors may contribute to causing delay in recognition and diagnosis of PCS in the emergency context. This narrative review approaches the issue of diagnostic error in PCS from different perspectives, including anatomical and demographic considerations as well as pitfalls and problems associated with various stages of prehospital and emergency department assessment. Strategies and approaches to improve speed and accuracy of recognition and early management of PCS are outlined.

## Introduction

Physicians long viewed posterior circulation stroke (PCS) as an entity sufficiently distinct from anterior circulation stroke (ACS) to justify focusing on particulars of management rather than attempting to identify stroke etiology and deriving therapeutic recommendations (1). The initiation of the New England Medical Center Posterior Circulation Registry (NEMC PCR) in 1988 constituted a critical turning point, as this research provided a large body of new clinical and imaging information which challenged this historical view and emphasized that PCS and ACS were, in fact, more alike than they were different. In the wake of this work, the number of publications dealing with a wide range of PCS-related topics increased dramatically. Nevertheless, despite advancing knowledge about PCS, rates of misdiagnosis still exceed those in ACS, which is related to several functional-anatomical properties of the posterior circulation and the clinical consequences resulting from acute vascular pathology. These inherent characteristics furthermore lead to several challenges concerning the correct recognition and diagnosis of PCS in the emergency department.

## Why PCS Poses a Challenge to Correct Diagnosis

### Differences in Vascular Anatomy and Susceptibility to Pathology

While the general nature of stroke in the anterior and posterior circulation is similar in many respects, there are distinct anatomical differences between the carotid and the vertebrobasilar vascular anatomy contributing to some of the differences in the way PCS is conceptually approached. The posterior circulation consists of the vertebral arteries arising from the subclavian arteries, three paired cerebellar arteries, the basilar artery, and the posterior cerebral arteries. Unlike the internal carotid artery, which gives rise to many smaller branches, the bilateral vertebral arteries join to form one large single midline vessel, the basilar artery, which supplies the brainstem, occipital lobes, and thalamus. Vascular pathology of various kinds can lead to multi-level strokes in different anatomical regions of the posterior circulation (2, 3). Long circumferential arteries with a superficial course supply the lateral parts of the brainstem and the cerebellum, while small penetrating arteries direct blood to the medial portions of the brainstem and the base of the pons (4). In comparison to the anterior circulation, larger parts of the posterior circulation are fed by penetrating vessels with typical distributions of arterial supply. As these arteries do not form collaterals, vascular occlusion causes a lacunar stroke.

**Figure 1:**
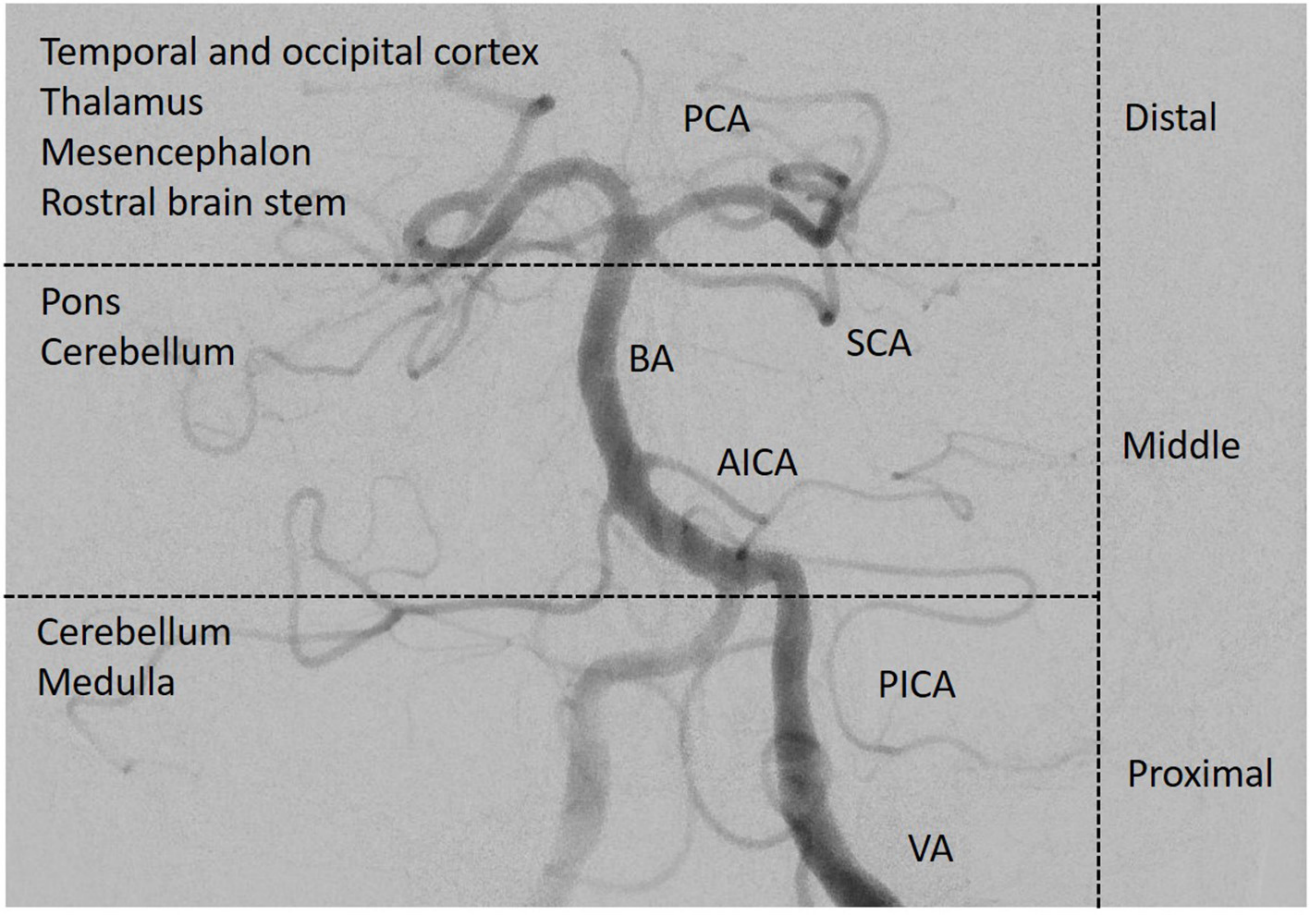
Posterior circulation vasculature. The vessels of the posterior circulation can cause multi-level strokes in different anatomical regions of the posterior circulation. The complexity of especially the structures in the brainstem makes localization of clinical signs and the site of infarction more difficult than in the anterior circulation. Angiography of the left vertebral and basilar artery. PCA, posterior cerebral artery; SCA, superior cerebellar artery; BA, basilar artery; AICA, anterior inferior cerebellar artery; PICA, posterior inferior cerebellar artery; VA, vertebral artery; distribution according to the New England Medical Center Posterior Circulation Stroke Registry (3). (*Image courtesy of C. Herweh, Frankfurt*).

The anatomical and functional complexity of the structures in the brainstem may make localization of clinical signs and identification of the site of infarction in the posterior circulation difficult. Most of the more recent posterior circulation stroke registries (5, 6) categorized stroke locations into the proximal, middle and distal vertebrobasilar artery territory as initially suggested by Caplan et al. (7) and demonstrated in Figure 1. In the NEMC PCR, most of the infarcts occurred in the distal territory (40%), followed by proximal (18%) and middle (16%) territory sites of infarction.

Atherosclerosis is the most common disease of the posterior circulation arteries. *In situ* thrombosis often leads to complete vessel occlusion, which in case of the basilar artery has devastating consequences with mortality rates of up to 90% (8). Embolism from the heart or proximal supplying vessels accounts for 20–30% of posterior circulation infarcts (9). Especially in young patients, vertebral artery dissections—due to trauma or hereditary disorders—can give rise to PCS. Small vessel disease often affects the paramedian branches of the basilar artery penetrating pontine tissue. While 40% of the brain's blood supply is provided by each internal carotid artery, ~20% of cerebral blood flow is attributable to the vertebrobasilar circulation (10). This predicts one out of five isolated cardioembolic strokes to be in the posterior circulation, as has been shown by diffusion-weighted MRI studies analyzing lesion patterns and stroke subtypes (9). The geometry of the vertebral artery origin from the subclavian artery differs compared to the carotid system since the vertebral artery has a nearly 90° take-off and is much smaller than its parent artery, thus increasing the risk factors for local atherosclerosis (11). Perhaps one of the most striking features of the vertebrobasilar circulation is the high frequency of anatomical variants—congenital anomalies, hypoplastic arteries, and adult retention of fetal arterial communications and patterns, to name the most relevant (12–14). Most are clinically insignificant, but some may impact stroke risks. For example, vertebral artery hypoplasia has been observed disproportionately frequently in strokes affecting the posterior inferior cerebellar artery (15), even though this has not been found to affect lesion size and clinical severity (16). In addition, knowledge about anatomical variants and anomalies in an individual may be relevant for identifying stroke etiology and the ensuing therapeutic consequences (17).

### Atypical Presentation as an Obstacle to Pre- and Early Intrahospital Symptom Awareness and Recognition

Positive outcome after ischemic stroke heavily relies on early treatment, which again depends on the fast and correct recognition and interpretation of stroke symptoms by patients and bystanders as well as by emergency medical service (EMS) and emergency department (ED) staff in both the pre- and early intrahospital phase. In this context, less classic or less commonly-known symptoms and atypical patient characteristics may represent specific challenges to PCS identification.

#### Lower Awareness for PCS Signs and Symptoms

**Figure 2:**
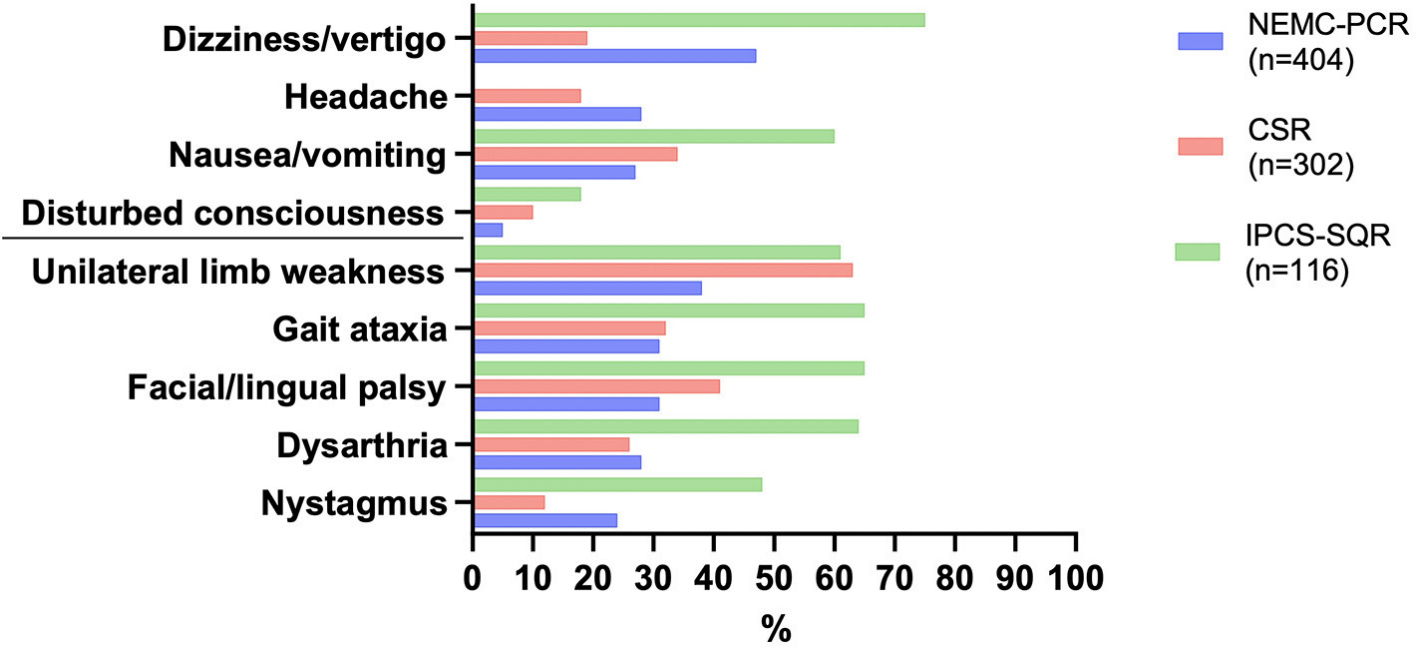
Most common symptoms in posterior circulation stroke as reported in the three large registries. NEMC-PCR, New England Medical Center Posterior Circulation Registry (23); CSR, Chengdu Stroke Registry (24); IPCS-SQR, Ischaemic Posterior Circulation Stroke in the state of Qatar Registry (25).

A high level of public awareness of stroke symptoms and the need to seek immediate medical attention is crucial for effective acute stroke treatment. Although no study has specifically focused on signs of PCS, research indicates that overall, there is much room for improvement. A study focusing on temporal trends in public awareness between 1995 and 2005 in Cincinnati found that knowledge of stroke warning signs only slightly improved: those able to name three warning signs rose from 5 to 16%, while there was no improvement in the ability of the public to name at least one warning sign (18). Not surprisingly, of typical stroke symptoms, the one named least frequently was trouble seeing/visual impairment. Interestingly, visual field abnormalities are among the most common manifestations of PCS yet constitute a symptom of which patients are often unaware (19). Finally, a Korean survey noted an underappreciation of stroke warning signs other than sudden paresis or numbness (20). Subsequently, it is not surprising that process times like onset-to-door and door-to-imaging times are significantly higher for PCS (21). A recent systematic review aimed to identify the characteristics of acute stroke presentations associated with inaccurate identification by EMS (22). The authors conclude from data reported in 21 studies that between 2 and 52% of all stroke presentations transported by EMS are not diagnosed on-site. The most common stroke presentations in these cases included posterior circulation symptoms such as nausea/vomiting, dizziness, and visual disturbance/impairment. Clinical manifestations of PCS and differential diagnoses to consider are presented in Table 1, Figure 2. While present in patients with an acute stroke, most frequently in those with PCS, these symptoms may occur in a wide range of conditions and thus possess a low signal-to-noise ratio when it comes to stroke detection. Mental status alterations—a term way too imprecise for a wide variety of cognitive and behavioral symptoms reported in PCS—have been reported in up to 25% of missed stroke cases (26–28). However, due to the anatomical features and idiosyncrasies discussed above, it is essential to recognize that these symptoms rarely occur in an isolated fashion in acute stroke. PCS can present with a wide range and combination of symptoms and signs, some of which overlap with those caused by ACS.

**Table 1:** Clinical manifestations of posterior circulation stroke.

**Territory**	**Affected territory**	**Clinical manifestation**
Distal	Posterior cerebral artery Top of the basilar artery	Occipital cortex: visual field defect with contralateral homonymous hemianopia, photopsia, and visual illusion; bilateral: cortical blindness, amnesia and agitation (Anton's syndrome) Thalamus: impairment of arousal and orientation, learning and memory, personality, and executive function; contralateral hemisensory loss, hemiparesis and hemiataxia, and pain syndromes, visual field deficits, sensory loss, weakness, and dystonia left: language deficits; right: visual-spatial deficits Mesencephalon, thalamus and occipital and temporal lobe: unconsciousness, oculomotor disturbances, cortical blindness, neuropsychological and mnestic deficits
Middle	Common brainstem syndromes	Weber's syndrome/paramedian and lateral midbrain infarct: ipsilateral III nerve palsy, contralateral hemiplegia Foville's syndrome/pontine tegmentum: Unilateral horizontal-gaze palsy, contralateral hemiparesis Wallenberg's syndrome/lateral medullary infarct: ataxia, vertigo, nystagmus, nausea and vomiting, loss of pick sensation in the ipsilsateral side of the face and contralateral side of the body, dysphagia, dysarthria, ipsilateral Horner's syndrome
Proximal	Superior cerebellar artery (from upper basilar artery) Posterior inferior cerebellar artery (from intracranial vertebral artery) Anterior inferior cerebellar artery (from lower basilar artery)	Ipsilateral: limb dysmetria, Horner's syndrome; contralateral: loss of sensation for temperature and pain, IV nerve palsy, hearing loss, sleep disorder When infarct spares the medulla: vertigo, headache, gait ataxia, appendicular ataxia, horizontal nystagmus, with medullary involvement: Wallenberg's syndrome Vertigo, vomiting, tinnitus, dysarthria, dysphagia, Ipsilateral conjugate-lateral gaze palsy Ipsilateral: Limb motor weakness, facial palsy, hearing loss, trigeminal sensory loss, Horner's syndrome, appendicular dysmetria

*Differential diagnosis of posterior circulation stroke: intoxication, infectious disorders, posterior reversible encephalopathy syndrome, migraine, seizure, benign paroxysmal peripheral vertigo, Meniere's disease, Wernicke's encephalopathy, central pontine myelinolysis, electrolytse disturbances*

As PCSs often present with non-specific symptoms such as dizziness, headache, nausea, and vomiting (2, 24), these are usually not interpreted as potential stroke symptoms by prehospital care providers and subsequently not assessed in this context. On the contrary, Andersson et al. (29) found that precisely those symptoms were more frequently documented and evaluated in patients in whom no stroke was suspected. This is extremely important to acknowledge in particular because the framing of a call as a potential stroke significantly impacts emergency department processes. The positive impact of early stroke identification and ED pre-notification in general (30) may generate a false sense of security with ED personnel over-relying on EMS staff's diagnostic impression and decision-making (31). Similarly, widely-used triage tools have been shown to under-appreciate the idiosyncrasies of neurological emergencies (32, 33). Atypical stroke symptoms may not only obscure subtler neurological abnormality, but they may also make the clinical assessment, especially by non-neurologists, difficult. Not surprisingly, there are also reports showing that clinical deficits in hyperacute stroke assumed to be caused by pathology in the anterior circulation eventually turn out to be PCS (34). Localizing capacities are thus brought to their limits, which would not be worrisome if a stroke is recognized as such and the necessary diagnostic and therapeutic measures ensue. All of the challenges mentioned above contribute to a lower likelihood of early arrival of PCS patients in the ED (35) and more frequent delays in neurological evaluation after initial ED assessment and delayed intravenous tissue plasminogen activator administration compared with ACS patients (36).

Recent studies indicate that 20–60% of acute ischemic strokes are missed in the emergency room setting (37, 38). Of these, PCSs are nearly three times more likely than ACSs to be missed, especially when presenting with nausea/vomiting and dizziness (37). The risk of misdiagnosis is high when presenting complaints are mild, non-specific, or transient, suggesting that many cases of diagnostic error relate to symptom-specific factors and perceived degree of impairment (38). While these data refer to general ED populations, stroke is even less frequently suspected in the young due to the lack of cardiovascular risk factors and a different range of potential etiologies. According to a recent study, these aspects underlie about 30% of missed strokes in young patients in the ED (39). Clinical signs that were initially missed in 50% of patients later identified by the first neurological consultation included Horner's syndrome, mild focal weakness (monoparesis or hemiparesis), ataxia, nystagmus, and hemianopia. Misdiagnosed patients were more frequently females, had a significantly higher prevalence of dissections and stroke involving the posterior circulation. Another study found that patients aged 35 years or below with PCS were more likely to be misdiagnosed (40). An especially vulnerable population are women: several studies found that women present more often with atypical stroke symptoms than men (39, 41). This situation is made even more difficult because there is a higher incidence of benign causes of symptoms such as headache or vertigo in women and that several stroke mimics share these characteristics with stroke chameleons, i.e., atypical stroke presentations (42, 43).

#### Shortcomings of Pre- and Early Intrahospital Scales and Tools

Different instruments for rapid stroke recognition have been developed, most of these predominantly intended for prehospital assessment by EMS personnel. The Face Arm Speech Test (FAST) is perhaps the most popular, also designed to aid stroke sign recognition by the general public. Prehospital stroke detection scales have been found to have similar shortcomings, with e.g., FAST missing about half of PCS (44, 45). Furthermore, patients with stroke misdiagnosis were commonly FAST-negative with non-specific symptoms including altered mental status, dizziness, and nausea/vomiting often associated with PCS, a finding that provides a false sense of security during ED assessment (46). In addition, recent years have seen a relative predominance of research concerning the suitability of prehospital stroke scales to recognize patients with large-vessel occlusion, who—as potential candidates for endovascular therapy (EVT)—require fast allocation to an EVT-capable stroke center (47). The primary focus here has been the detection of anterior circulation pathology rather than consideration of a subgroup of stroke patients with atypical symptoms and less-clear long-term benefit from acute interventions.

The National Institutes of Health Stroke Scale (NIHSS) is the most widely used deficit rating scale for assessing patients with acute ischemic stroke. While it has been shown to have a significant association with vessel occlusions in patients with ACS, performance in patients with PCS is poorer (48). Accordingly, PCS patients from the Acute Stroke Registry and Analysis of Lausanne had lower NIHSS at admission than ACS patients (49). The vast majority of PCS patients have a baseline NIHSS scores ≤4 (50), and even a value of 0 cannot rule out the presence of stroke, a finding reported in PCS patients in particular. In those patients commonly presenting with symptoms like headache, vertigo, and nausea and truncal ataxia as the most common neurologic signs (51), the NIHSS drastically underestimates the degree of stroke-associated functional impairment.

### The Risk of False-Negative Neuroimaging of the Posterior Fossa

Brain imaging plays a pivotal role in the differential diagnosis of neurological deficits, and CT is usually employed in the emergency setting because of its wide availability and speed of the examination. Due to bone-related artifacts and suboptimal brainstem resolution, however, the ability of this imaging modality to visualize small—in particular pontine and medullar—lesions is limited. Studies suggest that the sensitivity of CT for the detection of acute PCS is low (52) and that a negative CT may lead to false reassurance and missed stroke diagnoses in the emergency setting, especially in patients with less severe or inconclusive symptoms (53). To some extent, this disadvantage is attenuated when multimodal CT-imaging (CT angiography and CT perfusion) is employed, as reported for patients with acute vestibular syndrome who received intravenous thrombolysis triggered by information supplied by these procedures (54). One study found that while there were lower rates of early ischemic signs on admission CT and overall arterial pathology in PCS than in ACS, intracranial arterial pathology was more prevalent in the former (49). On a related note, in certain constellations of high clinical certainty of an acute cerebrovascular event, CT angiography is mandatory for demonstrating the site of vascular occlusion, thereby guiding treatment decisions (55). Compared to digital subtraction angiography (DSA), CT angiography is a reliable method for detecting lesions in the posterior circulation. It may, due to its relative ease of applicability, frequently be used instead of DSA. Similarly, adding CT perfusion to the scanning protocol may improve diagnostic accuracy (56). However, particularly in vertebral artery imaging, DSA remains superior (57).

Diffusion-weighted MRI (DWI) was introduced and established as a routine imaging procedure in acute ischemic stroke in the late 1990s; since then, many studies covering numerous different facets of ischemic stroke diagnostics have been published. DWI is exquisitely sensitive and able to demonstrate even minutely-sized acute ischemic lesions (58). The impaired mobility of water protons in ischemic tissue generates a strong signal against the background of healthy tissue on DWI, which provides high contrast of the lesion. The characterization of especially brainstem ischemic stroke lesions via imaging—previously only possible in post-mortem neuroanatomical studies—has since seen tremendous improvement (59). The number of publications dealing with routine clinical use of DWI related to specific aspects of PCS has risen substantially, and various clinical-anatomical facets have been explored (60, 61). However, despite the obvious advantages of DWI, a considerable number of infarcts may still be missed in cases of false-negative imaging (62), which was reported in the context of small lacunar lesions (63), in association with minor clinical deficits of <5 NIHSS points (64), and when MRI was performed very shortly after symptom onset (65). In addition, false negativity of DWI was found to occur five times more often in PCS (66). This phenomenon has been attributed to a different temporal evolution of DWI hyperintensities in acute brainstem infarcts compared to hemispheric stroke in the anterior circulation (67). As sensitivity increases over time, an early negative MRI, in particular, should not be relied upon too readily to rule out PCS, especially when symptoms persist.

## Diagnostic Error in the Emergency Context

Diagnostic error constitutes a substantial hazard to patient safety, and its potential consequences such as permanent disability or death are dire (68). It disproportionally affects neurological disorders and cerebrovascular events like stroke in particular (38, 41, 69–71). As a result, time-sensitive treatments may not be administered, and established standards of stroke care or secondary preventive measures may not be implemented. These missed opportunities bear significant medical and socioeconomic ramifications like higher rates of disability and mortality (72), higher hospital readmission (37), and prolonged hospitalization (70).

Bedside examination and clinical reasoning and decision-making are particularly prone to error (73, 74). In the latter two, clinicians employ heuristics in order to process complex information and plan work-up and treatment efficiently. They are indispensable in day-to-day practice, but in particular in the prehospital and emergency department context, which are fast-paced environments where there is often only limited or incomplete information available upon which part of the diagnostic considerations are based. In addition, time and resource constraints, frequent interruptions, and the need to multitask characterize these workplaces. Despite their undeniable value, heuristics are associated with certain pitfalls, which may lead to diagnostic error (75, 76). Accordingly, failed heuristics have been identified as one type of cognitive error occurring in the ED (77). Some of the diagnostic challenges presented by PCS and discussed above may be linked to different kinds of cognitive errors, such as diagnostic anchoring when EMS staff initially do not consider stroke, and later it is not introduced into the spectrum of differential diagnoses. Similarly, false reassurance by a negative CT scan can be considered an instance of blind obedience (76). These heuristics need to be viewed in the context of two different modes of information processing and management, a Type 1 “intuitive” and a Type 2 “analytical” mode of thinking, each of them possessing distinct merits and weaknesses (78). A number of strategies and interventions have been suggested to address these cognitive factors and the employment of Type 1 and Type 2 thinking, e.g., through debiasing techniques, reflective practice, or cross-checks. However, evidence for their effectiveness especially in the emergency care system is limited (79). There are no initiatives directly addressing cognitive errors in missed diagnoses of stroke in general and PCS in particular but a variety of solutions targeting different stages of the process of recognizing and diagnosing stroke have been suggested, and both implicit and explicit reverberations of cognitive phenomena and corresponding corrective strategies can be identified therein.

## Approaches to Solving the Problem of PSC Misdiagnosis

### Improving Symptom Recognition Prehospitally and During Triage

Timely recognition of stroke symptoms in the prehospital context as the first link in the chain of acute stroke care is an essential precondition for all following phases and refinement efforts. The need for improvement here is underscored by the fact that onset-to-door times have seen comparatively little change in comparison to intrahospital process times (30, 80, 81). Campaigns targeted at raising public stroke awareness may aid in increasing knowledge about stroke symptoms and the subsequent motivation to seek medical advice (82), even though help-seeking behavior has been found to be more dependent on perceived symptom severity than on actual symptom knowledge (83). The dominant representation of motor and speech disturbances in many public campaigns and the more pronounced functional impairment frequently associated with them may further increase disparities regarding the appropriate recognition and interpretation of atypical stroke symptoms or mild deficits. One challenge to address in the future will be to adequately represent these stroke manifestations without sacrificing brevity and memorability for application in public incentives. One of these respective attempts concerns the extension of the FAST mnemonic to include an assessment of balance and eye movement abnormalities, BE-FAST (84). Despite the lack of prospective studies, this modification of a screening method used by laypersons as well as EMS dispatchers and providers alike may be a promising strategy to pursue. In a retrospective study, BE-FAST was found to be a very sensitive tool for screening among hospitalized patients evaluated through an inpatient stroke alert system (85). Even though shortcomings of preclinical stroke screening instruments regarding PCS diagnosis have been appreciated, there have been relatively few efforts to supplement them with additional tools for PCS recognition (86). The same holds for severity scales like the NIHSS, for which an extended version, the eNIHSS, appreciating the posterior circulation has been offered (87) but does not appear to have gained much practical traction. Increasing knowledge and awareness in EMS staff regarding atypical stroke syndromes as those frequently found in PCS will be an important target for future work to reduce prehospital delays and errors in the early stages of patient assessment and allocation. One ambulance service in the UK added nausea to their prehospital stroke screening tool, which also includes vertigo, visual problems, and ataxia as further signs indicative of PCS (88). Another study demonstrated that an initiative as simple as training paramedics to perform the finger-to-nose test may facilitate PCS identification (89). The particular relevance of such efforts is also emphasized in the context of a recent study suggesting that ED staff does appear to rely on EMS staff's diagnostic impression (31). Hence, when EMSs fail to recognize stroke and do not pre-notify the ED, ED processes are negatively impacted. It follows that triage nurses are another important target population for initiatives aimed at increasing knowledge about and awareness of atypical stroke presentations. With regard to the shortcomings of established triage instruments, these may either be complemented by a neurological assessment, or dedicated neurological triage instruments (90) may be applied. In addition, the use of “do not-to-miss” diagnoses checklists for common complaints such as headache or dizziness has been advocated (91, 92), and their potential impact on ED diagnostic quality and processes deserves further prospective exploration.

### Strategies to Improve Diagnostic Yield in ED Clinical Assessment and Imaging

Considerable efforts have been devoted to improving the diagnostic accuracy of patients presenting with vertigo. In view of the costs caused by overdiagnosis and overtreatment of benign causes of dizziness as well as inadequate use of diagnostic methods in the diagnosis of stroke, in particular imaging, a sensitive yet quick and cost-effective assessment of patients with vertigo is much needed (93). In this regard, much attention has been drawn to an improved approach to history taking focusing on timing and triggers rather than symptom quality (94, 95), allowing for categorization of vestibular syndromes as either acute, triggered-episodic, spontaneous-episodic, or chronic, and the development of clinical pathways and algorithms to differentiate potential etiologies and guide an adequate syndrome-specific work-up (96). The HINTS (head impulse test, nystagmus, test of skew) diagnostic triad has been extensively investigated (97), and several modifications such as additional bedside assessment of hearing (98) or ataxia (99) have been proposed. Importantly, the head impulse test (HIT) as an essential component of these targeted forms of examination is underutilized in the ED: one study (100) found it was applied to patients with dizziness in only 5% of cases and in ~7% of cases with acute vestibular syndrome, for which it is most suited. This is all the more relevant since appropriately trained ED physicians are able to accurately administer the assessment (101). To reduce inter-observer variability and increase reliability, the test may be performed using video goggles, allowing for quantification of vestibular function and skew deviation (97). Such a procedure is assessed in an ongoing multicenter phase II trial, the AVERT (Acute Video-Oculography for Vertigo in Emergency Rooms for Rapid Triage) trial (102). With some of the available systems providing feedback regarding the correct velocity of a given impulse, their usefulness in the ED setting with examiners from different levels of skill and experience becomes immediately evident. Further development of this technology is underway, aiming at making its application more feasible and user-friendly in the ED setting (103). Automated saccade analysis may usefully complement video-oculography based HIT (104).

Whether or not the presence of a neurologist is necessary for reducing the rate of diagnostic error on PCS is equivocal: The presence of in-house neurology residents was associated with a lower risk of missed stroke in young patients but only after the exclusion of those patients who did not receive an emergency neurological consultation (105). However, even if a specialist assessment is obtained, the risk of missing the correct diagnosis is not fully abolished (72). In addition, community and academic hospitals, usually with easier access to neurological expertise in the latter, did not differ in the rate of missed strokes (37). Targeted education of neurology and ED physician trainees working in the ED concerning atypical stroke presentations may hence be an opportunity to further reduce diagnostic error in the ED. If direct neurological consultation is neither possible nor feasible, technology enables the remote assessment of patients with suspected stroke (106) and a wide variety of neurological conditions. Dizziness and vertigo have also been targets of telemedical approaches (107). Connected technology for data acquisition in conjunction with information from the patient's history and imaging may feed into the development of machine learning-based decision support solutions (108). It finally bears mentioning that the ongoing COVID-19 pandemic has substantially boosted the need for efforts to improve remote assessment and management of patients with these complaints (109, 110).

Regarding the pitfalls associated with MRI imaging in case of suspected PCS, several strategies may be pursued, such as adjusting MRI sequences with regard to slice thickness and orientation (111), using higher b-values for better contrast (112), adding additional perfusion sequences (113), or performing MRI in a time window of 5–12 h after symptom onset for increased sensitivity (59). Many argue that despite higher diagnostic accuracy of MRI, it commonly involves complex workflows that could potentially cause treatment delays and that performing comprehensive CT at presentation is the most cost-effective initial imaging strategy at comprehensive stroke centers (114). Even in light of these important areas of limitations and discordance, increased use of DWI in patients with atypical or unspecific symptoms in the ED is an especially useful aid in diagnosing entities such as cerebellar stroke presenting with isolated vertigo (115) and in evaluating patients with symptoms suspected to be stroke mimics (116), or those with migrainous stroke (117). MRI, therefore, plays a pivotal role in guiding the correct diagnosis and treatment of patients with PCS. In this regard, the formulation of imaging guidelines for patients presenting with atypical symptoms is an important area to focus on to further improve diagnostic accuracy and yield, particularly with respect to PCS (118)—all the more so since current recommendations emphasize symptom duration and patient selection for different therapeutic options—again with a focus on the anterior circulation (119, 120).

**Figure 3:**
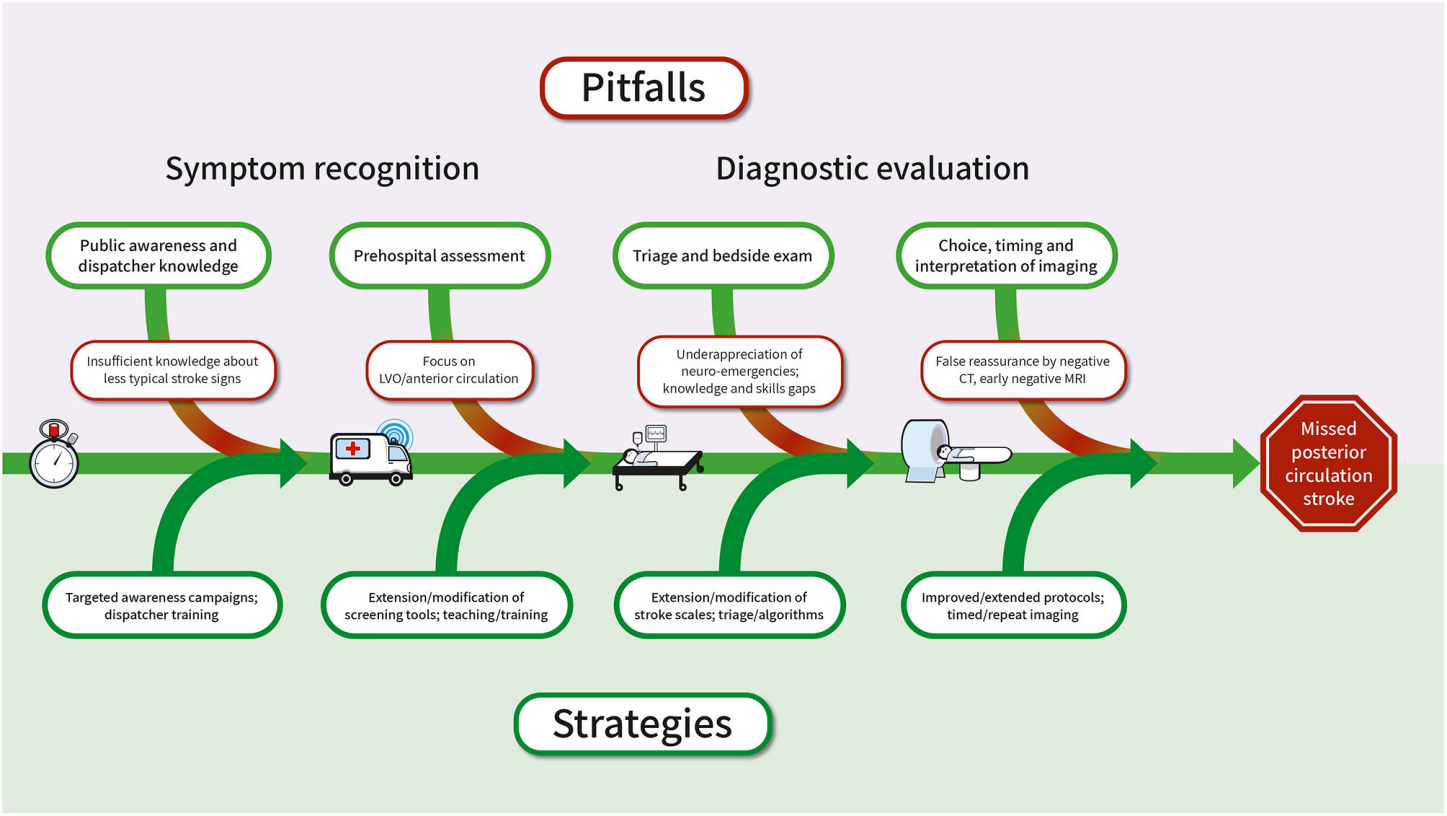
Pitfalls associated with the diagnosis of PCS in the chain of acute stroke care and suggested approaches to solution. CT, computed tomography; MRI, magnetic resonance imaging; PCS, posterior circulation stroke.

Figure 3 summarizes pitfalls and challenges and approaches to overcome them with respect to the early links in the chain of acute stroke care.

### Challenges and Opportunities for PCS Diagnostic Accuracy in the Context of the Coronavirus Disease 2019 (COVID-19) Pandemic

The ongoing COVID-19 pandemic has been posing extraordinary challenges to medicine and healthcare. The surge of infections in particular during the first wave of the pandemic frequently necessitated the reorganization and restructuring of prehospital and emergency room pathways of stroke patients and the reallocation of resources, impacting access diagnostics and therapy (121). Moreover, even in regions that were not as severely affected or where resources for acute stroke care were not limited, hospital admissions for cerebrovascular events decreased, presumably reflecting the influence of social distancing measures (122, 123). Not only may these cause patients to not seek medical help in the first place but they may theoretically impede the clinical assessment (124). Hence, there has been growing need for efforts to improve remote evaluation and management of patients with neurologic complaints. The use and acceptance of teleneurological consultations have been increasing (125, 126), and it is encouraging that observable neurological signs, which are feasible for remote assessment, appear to have better inter-rater reliability than elicitable signs, which often require direct contact with the patient (127). Since virtual HINTS and the Dix-Hallpike maneuver have been demonstrated to be applicable via telemedicine (110), solutions for additional components of the oculomotor exam may be developed and implemented. It remains to be seen, first, if and how these approaches, which can theoretically be applied to synchronous as well as asynchronous assessments, supplement or replace on-site examination, and second, how their implementation impacts on the diagnostic accuracy of PCS.

## Discussion

Emergency department utilization in many countries has substantially increased in recent years. The treatment of patients with neurological emergencies such as acute ischemic stroke is time-sensitive and requires swift action. In addition, the medical management of stroke patients today is more complex and multifaceted than ever before. The diagnostic process—an essential component of patient care in emergency departments—highly relies on successful teamwork among health care professionals, like EMS staff and ED healthcare teams, including physicians of various disciplines and nurses. This concerted and collaborative effort of all those participating in the acute management of stroke patients is critical to successfully circumnavigate the challenges and pitfalls of PCS diagnosis.

## Author Contributions

CH: conducted literature search, conceptualized review, and wrote the first draft. KS: conducted literature search and revised the manuscript. Both authors contributed to the article and approved the submitted version.

## Conflict of Interest

The authors declare that the research was conducted in the absence of any commercial or financial relationships that could be construed as a potential conflict of interest.
